# A Combined Approach to Women’s Health Is Associated With a Greater Likelihood of Repeat Mammography in a Population of Financially Disadvantaged Women

**Published:** 2007-09-15

**Authors:** Karen Y Gregory-Mercado, Julie Will, Susan True, Janet Royalty, E. Thomas Starcher, Olga Khavjou, William Helsel, William Kammerer, William Howe

**Affiliations:** CIGNA/Care Allies, Phoenix Health Facilitation Center. Dr. Gregory-Mercado was affiliated with the Division for Heart Disease and Stroke Prevention, National Center for Chronic Disease Prevention and Health Promotion, Centers for Disease Control and Prevention, Atlanta, Georgia, during the writing of this article.; Division for Heart Disease and Stroke Prevention, National Center for Chronic Disease Prevention and Health Promotion (NCCDPHP); Division of Cancer Prevention and Control, NCCDPHP; Division of Cancer Prevention and Control, NCCDPHP, Centers for Disease Control and Prevention, Atlanta, Georgia; McKing Consulting Corporation, Atlanta, Georgia; Research Triangle Institute International, Research Triangle Park, North Carolina; Information Management Services, Inc, Silver Spring, Maryland; Information Management Services, Inc, Silver Spring, Maryland; Information Management Services, Inc, Silver Spring, Maryland

## Abstract

**Introduction:**

Integrating one or more public health programs may improve the ability of programs to achieve common goals. Expanding knowledge on how program integration occurs, how it benefits each individual program, and how it contributes to the achievement of common goals is an important area of inquiry in public health.

**Methods:**

The National Breast and Cervical Cancer Early Detection Program (NBCCEDP) and the Well-Integrated Screening and Evaluation for Women Across the Nation (WISEWOMAN) program combined data from 10 of their overlapping state or tribal programs to calculate prevalence estimates of repeat mammography at 18 months. The data were stratified by whether women attended the combined program or only the NBCCEDP. Logistic regression analyses were conducted to identify factors that were thought to independently contribute to a greater likelihood of a woman receiving a repeat mammogram.

**Results:**

Women who participated in both programs were 1.5 to 5.1 times as likely to be rescreened, depending on program location, as women who participated only in the NBCCEDP. WISEWOMAN participants who received a follow-up WISEWOMAN screening for chronic disease risk factors within a year of their initial WISEWOMAN screening were 5 times more likely to return for a follow-up mammogram through the NBCCEDP than were WISEWOMAN participants who did not.

**Discussion:**

Participation in both the NBCCEDP and the WISEWOMAN program is associated with a greater likelihood of a woman returning for a follow-up mammogram within 18 months of her initial examination. Collecting more in-depth information on motivational factors and on the association between receipt of multiple services and a woman's engagement in a health program should be the subject of future research.

## Introduction

The Centers for Disease Control and Prevention (CDC) provides low-income, uninsured, and underserved women access to timely, high-quality screening and diagnostic services to detect breast and cervical cancer at the earliest stages through the National Breast and Cervical Cancer Early Detection Program (NBCCEDP) (1). In 1993, Congress authorized CDC to develop a combined approach to women's health by offering heart disease and stroke prevention services to women enrolled in the NBCCEDP.

The heart disease and stroke prevention program, later named the Well-Integrated Screening and Evaluation for Women Across the Nation (WISEWOMAN) program, was first funded in 1995 (2). Low-income women aged 40 to 64 years enrolled in the NBCCEDP are qualified for WISEWOMAN.

Many health interventions include testing for cholesterol, blood glucose, and blood pressure, but WISEWOMAN also provides lifestyle intervention and referral services. Through WISEWOMAN, qualified women receive chronic disease risk factor screening and health education interventions that help them lower their risk for heart disease. By helping women adopt healthy eating patterns and encouraging them to be more physically active, WISEWOMAN aims to help women know their risk for heart disease and develop a heart-healthy lifestyle.

The coordination of these two programs is one example of an integrated and comprehensive approach to improving the public's health. The successful implementation of such an approach, however, requires comprehensive changes to health care systems (3). Understanding how the coordination of multiple chronic disease programs might enhance the effectiveness of each is key to advancing prevention efforts. As part of an overall assessment on the benefits of combined approaches to preventive health, WISEWOMAN and the NBCCEDP are conducting analyses to explore these relationships. This report describes the results of one part of those analyses: a comparison of breast cancer rescreening rates among NBCCEDP participants who also participated in the WISEWOMAN program with rescreening rates among NBCCEDP participants who did not.

## Methods

### NBCCEDP and WISEWOMAN

The NBCCEDP is implemented through cooperative agreements with grantee programs in all 50 states, the District of Columbia, 4 territories, and 13 American Indian and Alaska Native jurisdictions. WISEWOMAN is currently implemented in 13 state health departments and 2 tribal organizations. Participation in WISEWOMAN is limited to women enrolled in the NBCCEDP, although not all eligible NBCCEDP participants choose to participate in WISEWOMAN, and not all NBCCEDP sites offer the WISEWOMAN program.

Twice a year, WISEWOMAN and NBCCEDP grantees send a report to CDC on the clinical services provided, including data on participant demographics, clinical procedures and outcomes, and physiologic measures. This report is based on data from 10 state or tribal grantees that implemented both WISEWOMAN and NBCCEDP and had enough participants during the study period to produce stable estimates of follow-up mammography screening rates.

From 2000 to 2004, 17% of NBCCEDP participants at the 10 grantee locations (range, 8%–39%) were also enrolled in WISEWOMAN. The study cohort consisted of women enrolled in the NBCCEDP from January 1, 2000 (the date of the first WISEWOMAN screening) through June 30, 2003. Women who had a normal result on an initial mammography screening during this period were followed for 18 months to determine whether they received a routine rescreening mammogram through the NBCCEDP. A woman was considered to be a participant in both programs if she received a WISEWOMAN disease risk factor screening within 18 months of her initial NBCCEDP mammography screening.

Rescreening rates for each of the 10 grantee locations were weighted by each location's sample size to produce an overall estimate of NBCCEDP rescreening rates by WISEWOMAN participation. A fully adjusted model was used to control for demographic characteristics that may be associated with rescreening rates (4,5), such as age, race/ethnicity, and program location. An additional analysis of NBCCEDP rescreening rates among women who participated in both WISEWOMAN and the NBCCEDP also controlled for characteristics including education, baseline health status, participation in WISEWOMAN lifestyle interventions, and whether they had been rescreened for chronic disease risk factors through the WISEWOMAN program within a year of their initial WISEWOMAN chronic disease risk factors screening.

The first analytic cohort consisted of 68,522 individuals who received a baseline mammogram during the enrollment period.

## Results

Across all 10 programs, participation in both WISEWOMAN and the NBCCEDP was associated with a higher percentage of follow-up mammograms, with adjusted odds ratios ranging from 1.5 to 5.1 (Table 1). Overall, women who participated in both the NBCCEDP and the WISEWOMAN program were 2.8 times more likely to have received a follow-up mammogram than were women who participated in the NBCCEDP alone (Table 2). The demographic characteristics associated with an adjusted greater likelihood of having had a repeat mammogram were being aged 50 or older and being white (Table 2).

Among the 13,742 women who participated in both programs, certain characteristics increased the likelihood that women received a follow-up mammography screening. Rescreening rates were significantly higher among those older than 49 years. American Indian or Alaska Native women and women of unknown races had higher rates than did whites, blacks, Hispanics, and Asians. Nonsmokers were more likely to be rescreened than were smokers. Those who received follow-up WISEWOMAN screening services within a year of their initial WISEWOMAN screening were more likely than those who did not to receive a mammography rescreening  (Table 3). Adjusted results indicate that WISEWOMAN participants who received a follow-up WISEWOMAN screening within a year of their initial WISEWOMAN screening were 5 times more likely to return for a follow-up NBCCEDP mammogram than were WISEWOMAN participants who did not.

## Discussion

Excluding nonmelanoma skin cancer, breast cancer is the most common form of cancer among U.S. women, and it accounts for a third of newly diagnosed cancer cases among women each year (6). Although appropriate preventive services, including mammography screening, have been shown to reduce the number of cancer deaths (7), mammography screening is underused (7,8). Estimates of the percentage of women who have undergone mammography screening range from 32% to 91%, while estimates of the percentage that have undergone rescreening range from 28% to 92% (4,9). However, estimated screening rates are substantially lower among financially disadvantaged populations, such as those served by the NBCCEDP and WISEWOMAN (4,5). In an NBCCEDP study of rescreening rates at selected sites (which reflected rescreenings that program participants reported receiving outside the NBCCEDP as well as program-delivered rescreenings), average rates were as high as 72.4% after 18 months and 81.5% after 30 months; however, rescreening rates were lower in the most disadvantaged populations — women who were older, were of racial/ethnic minorities, and had lower levels of education (9).

Although the rescreening rates among women in this cohort who were older, white, American Indian or Alaska Native, or WISEWOMAN participants are encouraging, the overall results show that practices and strategies to increase rescreening rates among underserved women need to be developed. The chances that a patient will follow cancer screening recommendations are influenced both by personal factors and by the patient's health care environment. Thus, understanding the interplay of these factors is critical for the development of effective cancer prevention programs (3).

Joint participation in the NBCCEDP and the WISEWOMAN programs may be associated with higher cancer rescreening rates for at least three reasons. First, women in the NBCCEDP who choose to participate also in WISEWOMAN are likely to be more committed to their overall health care than those who choose not to participate (3). Second, women participating in both WISEWOMAN and the NBCCEDP may feel that they are receiving better, more comprehensive health care service and thus be more likely to return for follow-up screening as recommended (3,10). Finally, in addition to receiving annual reminders from the NBCCEDP about the importance of regular screenings for breast and cervical cancer, women who also participate in WISEWOMAN receive comprehensive cardiovascular disease screening and a series of culturally sensitive lifestyle interventions that provide additional contact with health care providers (10). In effect, WISEWOMAN reminder practices and multicomponent interventions may serve to reinforce the rescreening reminders that NBCCEDP participants receive, resulting in at least a doubling of the number of times that participants are prompted to be rescreened, a factor that might be critical for financially disadvantaged populations.

Findings from other studies also indicate that coordinated health care efforts have a synergistic effect. For example, in a meta-analysis, researchers found that interventions designed to increase older women's access to health care were more effective when used in conjunction with promotional strategies directed at individuals than access-enhancing strategies alone (11).

The findings in this report are subject to several limitations. The sample sizes for the variables listed in the tables differ because of missing data; however, missing data should have had little effect on the estimates presented here, given that the demographic profile of participants with missing data was not significantly different from that of participants with complete data. Mammography screening guidelines recommend annual or biennial screening for women aged 40 or older, whereas the rescreening rates reported here are based on a follow-up period of 18 months. Another limitation is that this study did not capture rescreening that NBCCEDP participants may have undergone outside of the NBCCEDP, nor did it adjust for differences in participants' mammography screening history or for the timeliness of historical screenings; these are all characteristics that might indicate other sources of funding for mammograms, as well as secular trends. Finally, women who are highly health-conscious may be more likely to participate in both WISEWOMAN and NBCCEDP and may be more likely to return for NBCCEDP rescreening.

Overcoming this self-selection bias in future NBCCEDP/WISEWOMAN studies will be difficult because, if the programs are fully integrated, women will have no choice but to attend both, effectively ensuring that motivation is not the key factor in attending. Thus, researchers will be unable to compare rescreening rates of women who attend both programs with the rates of those who attend only one program. Measuring health motivation and ensuring that all study participants are equally motivated might be another way to address this issue of self-selection bias. However, neither program currently has a measure of health motivation. Thus, the data provided in this study are the strongest available for these two programs.  

The results of this analysis suggest that participation in both the NBCCEDP and WISEWOMAN may result in better adherence to mammography rescreening recommendations. A number of likely explanations for these results have been proposed: women who receive multiple services may be more invested in the program or, alternatively, women who are more health-conscious may be likely to select a more intensive combined program. Consequently, further research is needed to better understand the reasons behind these findings.

## Figures and Tables

**Table 1:** NBCCEDP Participants Who Underwent Follow-up Mammography Screening Within 18 Months of Their Initial Mammograms, by Program Location and Participation in the WISEWOMAN Program, and the Increased Likelihood of Rescreening Associated with WISEWOMAN Participation, 2000–2004

Location	Program	No. Participants	Mammography		

			Rescreened, %	Unadjusted OR (95% CI)	Adjusted^a^ OR (95% CI)
A	NBCCEDP only	4,013	42	Ref	Ref
	NBCCEDP + WW	898	64	2.53 (2.18-2.94)	2.44 (2.09-2.86)
B	NBCCEDP alone	1,954	53	Ref	Ref
	NBCCEDP + WW	438	80	3.55 (2.77-4.55)	3.36 (2.61-4.34)
C	NBCCEDP alone	14,119	27	Ref	Ref
	NBCCEDP + WW	4,101	52	3.01 (2.81-3.24)	2.90 (2.69-3.12)
D	NBCCEDP alone	16,093	31	Ref	Ref
	NBCCEDP + WW	1,411	54	2.57 (2.31-2.87)	2.61 (2.30-2.96)
E	NBCCEDP alone	3,231	29	Ref	Ref
	NBCCEDP + WW	3,390	67	5.11 (4.60-5.68)	5.09 (4.58-5.66)
F	NBCCEDP alone	9,857	37	Ref	Ref
	NBCCEDP + WW	2,058	47	1.52 (1.38-1.67)	1.49 (1.35-1.64)
G	NBCCEDP alone	605	24	Ref	Ref
	NBCCEDP + WW	319	55	3.84 (2.87-5.12)	3.84 (2.84-5.18)
H	NBCCEDP alone	788	41	Ref	Ref
	NBCCEDP + WW	327	55	1.78 (1.37-2.30)	1.68 (1.29-2.21)
I	NBCCEDP alone	3,066	51	Ref	Ref
	NBCCEDP + WW	461	67	2.01 (1.63-2.47)	1.93 (1.57-2.38)
J	NBCCEDP alone	1,054	34	Ref	Ref
	NBCCEDP + WW	339	62	3.06 (2.38-3.94)	3.08 (2.39-3.97)

NBCCEDP indicates National Breast and Cervical Cancer Early Detection Program; WW, WISEWOMAN program; OR, odds ratio; CI, confidence interval; Ref, referent group.

a Adjusted for age and race/ethnicity.

**Table 2:** NBCCEDP Participants Who Underwent Follow-up Mammography Screening Within 18 Months of Their Initial Mammograms and Their Relative Likelihood of Doing So, by WISEWOMAN Participation, Age, and Race/Ethnicity, 2000–2004

Characteristic	No. Participants^a^	Mammography		

		Rescreened, %	Unadjusted OR (95% CI)	Adjusted^b^ OR (95% CI)
**Program**				
NBCCEDP alone	54,780	34	Ref	Ref
NBCCEDP + WW	13,742	58	2.71 (2.61-2.82)	2.75 (2.60-2.82)
**Age, y**				
40-49	30,614	28	Ref	Ref
50-59	30,000	46	2.13 (2.05-2.20)	2.14 (2.06-2.22)
≥60	7,767	52	2.71 (2.57-2.85)	2.78 (2.64-2.93)
**Race/ethnicity**				
White	39,795	41	Ref	Ref
Black	10,504	33	0.70 (0.67-0.73)	0.76 (0.72-0.80)
Hispanic	9,515	34	0.75 (0.72-0.79)	0.88 (0.83-0.92)
Asian^c^	1,866	28	0.57 (0.51-0.63)	0.65 (0.58-0.72)
American Indian or Alaska Native	5,133	46	1.25 (1.18-1.33)	0.63 (0.56-0.72)
Unknown	1,709	35	0.79 (0.71-0.87)	0.71 (0.63-0.79)

NBCCEDP indicates National Breast and Cervical Cancer Early Detection Program; WW, WISEWOMAN program; OR, odds ratio; CI, confidence interval; Ref, referent group.

a Participant totals differ because of missing data.

b Adjusted for participation at individual locations and in the WISEWOMAN program, and for age and race/ethnicity.

c Included women who identified themselves as Native Hawaiian or other Pacific Islander.

**Table 3:** Percentage of Participants in Both the NBCCEDP and the WISEWOMAN Program Who Underwent Follow-up Mammography Screening Within 18 Months of Their Initial Mammograms and Their Relative Likelihood of Doing So, by Participant Characteristics, 2000–2004

Characteristic	No. Participants^a^	Mammography		

		Rescreened, %	UnadjustedOR (95% CI)	Adjusted^b^ OR (95% CI)
**Sociodemographics**				
**Age, y**				
40-49	5,781	49	Ref	Ref
50-59	6,345	64	1.81 (1.68-1.94)	1.88 (1.72-2.06)
≥60	1,587	69	2.33 (2.07-2.63)	2.13 (1.84-2.46)
**Education**				
Less than high school	1,342	59	0.86 (0.76-0.97)	0.90 (0.77-1.05)
Some high school	1,304	59	0.86 (0.76-0.97)	0.86 (0.75-0.98)
High school graduate	5,422	63	Ref	Ref
Some college or more	4,058	56	0.77 (0.71-0.84)	0.76 (0.69-0.83)
**Race/ethnicity**				
White	9,096	59	Ref	Ref
Black	1,330	53	0.77 (0.69-0.87)	0.82 (0.71-0.94)
Hispanic	1,881	58	0.94 (0.85-1.04)	0.91 (0.79-1.04)
Asian^c^	402	46	0.60 (0.49-0.73)	0.68 (0.52-0.89)
AI or AN	781	61	1.10 (0.94-1.27)	1.27 (1.07-1.52)
Unknown	252	67	1.42 (1.09-1.85)	1.43 (1.05-1.95)
**Baseline health status**				
**Smoking**				
No	10,268	61	Ref	Ref
Yes	3,139	51	0.67 (0.61-0.72)	0.67 (0.61-0.74)
**Diabetes**				
No	12,429	58	Ref	Ref
Yes	1,310	58	1.00 (0.89-1.12)	0.91 (0.79-1.05)
**Hypertension**				
No	8,266	57	Ref	Ref
Yes	4,768	60	1.16 (1.08-1.25)	1.00 (0.91-1.10)
**High cholesterol**				
No	9,179	57	Ref	Ref
Yes	3,814	60	1.13 (1.05-1.22)	0.95 (0.86-1.04)
**Body mass index**				
<25.0	3,490	55	Ref	Ref
25.0-29.9	3,829	60	1.20 (1.10-1.32)	1.08 (0.97-1.20)
≥30.0	5,205	58	1.12 (1.03-1.22)	1.00 (0.90-1.11)
**WISEWOMAN characteristics**				
**Lifestyle intervention attendance**				
Zero sessions	4,098	57	Ref	Ref
One or more sessions	9,644	58	1.06 (0.98-1.14)	1.09 (1.00-1.20)
**WISEWOMAN rescreening at 10-14 months**				
No	9,385	47	Ref	Ref
Yes	4,357	81	4.83 (4.44-5.27)	5.02 (4.52-5.57)

NBCCEDP, National Breast and Cervical Cancer Early Detection Program; WW, WISEWOMAN program; OR, odds ratio; CI, confidence interval; AI, American Indian; AN, Alaska Native; Ref, referent group.

a Participant totals by characteristics differ because of missing data.

b Adjusted for all variables listed in the table, including WISEWOMAN rescreening.

c Included women who identified themselves as Native Hawaiian or other Pacific Islander.
